# RETRACTION: Comparison of the Postoperative Analgesic Effects of Paracetamol–Codeine Phosphate and Naproxen Sodium–Codeine Phosphate for Lumbar Disk Surgery

**DOI:** 10.1002/kjm2.12945

**Published:** 2025-01-15

## Abstract

Retraction: PolatR. , PekerK. , GülöksüzÇ. T. , ErgilJ. , AkkayaT. , “Comparison of the Postoperative Analgesic Effects of Paracetamol–Codeine Phosphate and Naproxen Sodium–Codeine Phosphate for Lumbar Disk Surgery,” Kaohsiung Journal of Medical Sciences 31, no. 9 (2015): 468–472. 10.1016/j.kjms.2015.07.001.26362959 PMC11915992

The above article, published online on 06 August 2015, in Wiley Online Library (wileyonlinelibrary.com), and has been retracted by agreement between the journal Editor‐in‐Chief, Wan‐Long Chuang; Kaohsiung Medical University; and John Wiley and Sons Australia, Ltd. In 2021, a third party raised concerns regarding evidence of unlikely similarity in the standard deviation values presented in Figures 1 and 2. The journal contacted the authors, who provided original data for evaluation by the journal. Following further correspondence, the journal and publisher concluded that the authors' response satisfied their concerns and that the investigation should be closed without further action.

In 2024, the complainant appealed to the journal and publisher to re‐open the investigation based on the substantial evidence of errors. The publisher accepted this appeal and re‐opened the investigation. The editors have conducted a review of the evidence and the provided dataset and found that the data includes substantial numbers of duplicated values across pairs of patients. The editors have determined that the substantial quantity of duplicated values is incompatible with a genuine randomized controlled trial and that the evidence compromises the conclusions of the article. As such, the parties agree that a retraction is necessary. The authors disagree with the retraction.

Retraction: R. Polat , K. Peker , Ç. T. Gülöksüz , J. Ergil , T. Akkaya , “Comparison of the Postoperative Analgesic Effects of Paracetamol–Codeine Phosphate and Naproxen Sodium–Codeine Phosphate for Lumbar Disk Surgery,” Kaohsiung Journal of Medical Sciences 31, no. 9 (2015): 468–472. 10.1016/j.kjms.2015.07.001.26362959 PMC11915992

The above article, published online on 06 August 2015, in Wiley Online Library (wileyonlinelibrary.com), and has been retracted by agreement between the journal Editor‐in‐Chief, Wan‐Long Chuang; Kaohsiung Medical University; and John Wiley and Sons Australia, Ltd. In 2021, a third party raised concerns regarding evidence of unlikely similarity in the standard deviation values presented in Figures 1 and 2. The journal contacted the authors, who provided original data for evaluation by the journal. Following further correspondence, the journal and publisher concluded that the authors' response satisfied their concerns and that the investigation should be closed without further action.

In 2024, the complainant appealed to the journal and publisher to re‐open the investigation based on the substantial evidence of errors. The publisher accepted this appeal and re‐opened the investigation. The editors have conducted a review of the evidence and the provided dataset and found that the data includes substantial numbers of duplicated values across pairs of patients. The editors have determined that the substantial quantity of duplicated values is incompatible with a genuine randomized controlled trial and that the evidence compromises the conclusions of the article. As such, the parties agree that a retraction is necessary. The authors disagree with the retraction.

